# Self-care interventions for sexual and reproductive health and rights for advancing universal health coverage

**DOI:** 10.1080/26410397.2020.1778610

**Published:** 2020-07-20

**Authors:** Manjulaa Narasimhan, Carmen H. Logie, Alice Gauntley, Rodolfo Gomez Ponce de Leon, Karima Gholbzouri, Nandi Siegfried, Heather Abela, Leopold Ouedraogo

**Affiliations:** a Scientist, Department of Sexual and Reproductive Health and Research; UNDP/UNFPA/UNICEF/WHO/World Bank Special Programme, World Health Organization, Geneva, Switzerland; b Associate Professor, Factor-Inwentash Faculty of Social Work, University of Toronto, Toronto, Canada; c Research Assistant, Factor-Inwentash Faculty of Social Work, University of Toronto, Toronto, Canada; d Sexual and Reproductive Health Regional Advisor, PAHO/AMRO, Montevideo, Uruguay; e Medical Officer, Women’s Reproductive Health, EMRO, Cairo, Egypt; f Independent Clinical Epidemiologist, Cape Town, South Africa; g Regional Advisor, Reproductive and Women's Health (RWH), AFRO, Brazzaville, Congo

**Keywords:** sexual and reproductive health, human rights, self-care interventions, universal health coverage, health delivery

## Abstract

WHO's normative guidance on self-care interventions for sexual and reproductive health and rights (SRHR) promotes comprehensive, integrated and people-centred approaches to health service delivery. Implementation of self-care interventions within the context of human rights, gender equality, and a life course approach, offers an underused opportunity to improve universal health coverage (UHC) for all. Results from an online global values and preferences survey provided lay persons' and healthcare providers' perspectives on access, acceptability, and implementation considerations. This analysis examines 326 qualitative responses to open-ended questions from healthcare providers (n = 242) and lay persons (n = 70) from 77 countries. Participants were mostly women (66.9%) and were from the Africa (34.5%), America (32.5%), South-East Asia (5.6%), European (19.8%), Eastern Mediterranean (4.8%), and Western Pacific regions (2.8%). Participants perceived multiple benefits of self-care interventions for SRHR, including: reduced exposure to stigma, discrimination and access barriers, increased confidentiality, empowerment, self-confidence, and informed decision-making. Concerns include insufficient knowledge, affordability, and possible side-effects. Implementation considerations highlighted the innovative approaches to linkages with health services. Introduction of self-care interventions is a paradigm shift in health care delivery bridging people and communities through primary health care to reach UHC. Self-care interventions can be leveraged by countries as gateways for reaching more people with quality, accessible and equitable services that is critical for achieving UHC. The survey results underscored the urgent need to reduce stigma and discrimination, increase access to and improve knowledge of self-care interventions for SRHR for laypersons and healthcare providers to advance SRHR.

## Résumé

Les directives normatives mondiales de l’OMS sur les interventions d’auto-prise en charge en matiere Santé sexuelle et reproductive et droits (SSRD) encouragent une approche globale des prestations de soins de santé, intégrée et centrée sur les personnes, dans leur environnement de vie quotidienne. La mise en œuvre des interventions d’auto-prise en charge dans le contexte des droits de l’homme, de l’égalité de genre et d’une approche basée sur le cycle de vie, offre une opportunité encore négligée d’élargir la couverture de la santé universelle (CSU). Les résultats d’une enquête en ligne sur les valeurs et les préférences mondiales ont montré les perspectives de particuliers et de professionnels de santé sur l’accès, l’acceptabilité et des considérations pour les interventions d’auto-prise en charge en matière de SSRD, notamment pour la mise en œuvre. Cette analyse examine les réponses qualitatives à des questions ouvertes qui ont été posées à 326 participants, notamment des prestataires de services de santé (n = 242) et des non-professionnels (n = 70) originaires de 77 pays. Les participants comptaient une majorité de femmes (66,9%) et venaient des régions de l’Afrique (34,5%), des Amériques (32,5%), de l’Europe (19,8%), de l’Asie du Sud-Est (5,6%), de la Méditerranée orientale (4,8%) et du Pacifique occidental (2,8%). Les participants étaient conscients des avantages multiples des interventions d’auto-prise en charge en matière de SSRD, notamment: l’exposition réduite à la stigmatisation et à la discrimination, la confidentialité accrue, moins d’obstacles à l’accès, l’autonomisation, la confiance en soi et une prise de décision éclairée. Leurs préoccupations comprenaient l’insuffisance de connaissances, la stigmatisation, l’accessibilité financière et les effets secondaires. Les considérations relatives à la mise en œuvre incluaient des approches novatrices pour les liens avec les services de santé, selon les besoins. L’introduction d’interventions d’auto-prise en charge de qualité est un véritable changement dans les prestations de soins de santé qui a le potentiel de desservir les personnes et les communautés par des soins de santé primaires en vue d’atteindre la CSU. Les interventions d’auto-prise en charge peuvent être utilisées par les pays comme passerelles leur permettant d’atteindre davantage de personnes avec les services de santé de qualité, accessibles et équitables qui sont essentiels pour mettre en œuvre la CSU. Les résultats de l'enquête ont souligné la nécessité urgente de réduire la stigmatisation et la discrimination, et améliorer la connaissance des interventions d'autosoins en matiere de la SSRD en particulier pour les professionnelles de santé et les prestataires en vue d'avancer SSRD.

## Resumen

La guía global de la OMS sobre las intervenciones de autocuidado en salud y derechos sexuales y reproductivos (SDSR) promueve un enfoque integral e integrado, centrado en las personas, donde la prestación de servicios de salud se encuentra en su entorno diario. La implementación de intervenciones de autocuidado dentro del contexto de derechos humanos, igualdad de género y enfoque del curso de vida, ofrece una oportunidad subutilizada de mejorar la cobertura universal de salud para todas las personas. Los resultados de una encuesta global en línea sobre valores y preferencias proporcionaron las perspectivas de las personas comunes y profesionales de salud acerca del acceso, la aceptabilidad y las consideraciones sobre las intervenciones de autocuidado para SDSR y su implementación. Este análisis examina las respuestas cualitativas a preguntas abiertas, que fueron proporcionadas por 326 participantes: profesionales de salud (n = 242) y personas comunes (n = 70) provenientes de 77 países. La mayoría de los participantes fueron mujeres (66.9%) provenientes de las regiones de África (34.5%), las Américas (32.5%), el sudeste de Asia (5.6%), Europa (19.8%), el Mediterráneo oriental (4.8%) y el Pacífico occidental (2.8%). Los participantes percibieron múltiples beneficios de las intervenciones de autocuidado para SDSR, por ejemplo: menor exposición al estigma y discriminación, mayor confidencialidad, menores barreras al acceso, empoderamiento, autoconfianza y una toma de decisiones informadas. Algunas de las preocupaciones mencionadas fueron insuficientes conocimientos, estigma, asequibilidad y efectos secundarios. Entre las consideraciones de implementación se describen enfoques innovadores en los vínculos con los servicios de salud, según fuera necesario. La introducción de intervenciones de autocuidado de calidad es un verdadero cambio de paradigma en la manera en que los servicios de salud son proporcionados, con gran potencial de crear puentes entre las personas y las comunidades por medio de los servicios de atención primaria para lograr cobertura universal de salud. Las intervenciones de autocuidado pueden ser aprovechadas por los países como vías para llegar a más personas con servicios de calidad accesibles y equitativos, lo cual es fundamental para lograr cobertura universal de salud. Los resultados de la encuesta subrayaron la urgente necesidad de reducir el estigma y la discriminación, ampliar el acceso y mejorar el conocimiento sobre las intervenciones de autocuidado para SDSR, a fin de que las personas comunes y profesionales de salud puedan promover la salud y los derechos sexuales y reproductivos.

## Introduction

Within ten years, the estimated global shortage of health workers to achieve and sustain Universal Health Coverage (UHC) is expected to grow to 18 million health workers.^1^ A fundamental shift in health service delivery is needed now to meet the sexual and reproductive health (SRH) needs, priorities and human rights of people and communities. While self care is conceptually not new, rapid advances in evidence-based technologies and products that can be accessed outside of the formal health sector increasingly acknowledge people as active participants in their own health, both as self-carers and care-givers.^2^ Definitions used by the World Health Organization (WHO) for self care and for self-care interventions are noted in Box 1.^2,3^ WHO’s global normative guidance on self-care interventions for sexual and reproductive health and rights (SRHR) promotes a comprehensive, integrated and people-centred approach to health service delivery that is within people’s everyday environment. Implementation of self-care interventions within the context of human rights, gender equality, and a life course approach, offers an underused opportunity to improve UHC for all.^4^

Box 1. WHO definition of self care and self-care interventions*

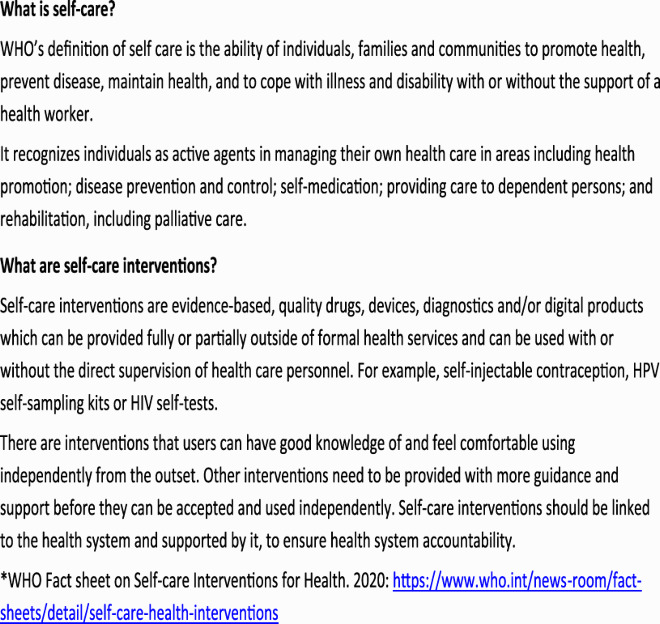

Several quality self-care interventions for SRHR, such as condoms and pregnancy tests, are already available in many settings. Others are being rapidly introduced, such as self-injectable contraception and self-sampling kits for human papilloma virus (HPV). Given that 232 million women in developing countries lack access to modern contraceptives and 300,000 women die each year from cervical cancer worldwide, a rights-based introduction of just these two interventions can have a significant impact for women and girls in low- and middle-income countries by providing them with additional choice and options to prevent or delay pregnancy and reduce morbidity and mortality from cervical cancer. Other available self-care interventions cover all key elements of the WHO global strategy on SRH,^5^ and many also hold promise to reach people living in remote areas, in humanitarian settings, or during health emergencies.^6,7^ Coupled with the increasing availability of digital technologies and mobile platforms, self-care interventions are essential tools for advancing health equity and access to realise UHC for SRHR.^8^

Self-care strategies can be framed as issues regarding *people-centred care,*^9^ whereby self care can be seen as a vehicle for empowerment and agency with a focus on capacity building.^10^ These approaches can also be understood from a *systems lens,*^4^ whereby self-care strategies are conceptualised as complementary to formal health care service provision and the focus is on optimising health outcomes via task shifting, ensuring linkage to care, and managing health.^11,12^ Self-care interventions should be supported by the health system, thereby ensuring that the health system remains accountable and can determine how to interact appropriately with and support implementation of these interventions. This is important given that many of these interventions are in various stages of development and availability and will require varying degrees and frequency of contact with different levels of the health system.^13^

The WHO conceptual framework for self-care interventions for health is grounded upon key principles of human rights, gender equality and a life course perspective, and considers the enabling environment as inclusive of trained healthcare providers, psychosocial support, education and information, access to justice, and freedom from violence, stigma and discrimination.^4^

People might choose self-care interventions for positive reasons, which may include convenience, cost, empowerment, a better fit with values or daily lifestyles, or the intervention may provide the desired options and choice. However, where there exist contexts of unequal power and potential for coercion or harm, people might also opt for self-care interventions to avoid the health system due to lack of quality (for instance, stigma from providers) or lack of access (for instance, in humanitarian settings). To ensure human rights are promoted and protected in the context of self-care interventions for SRHR, users must have information, autonomy and the ability to make decisions about their own lives and health. While this is true for all potential users of self-care interventions, it is particularly relevant for vulnerable populations including, but not limited to, sex workers, people living with HIV, Indigenous peoples, and transgender people, who face stigma, discrimination and violence in their efforts to secure their health and exercise their rights. Self-care interventions fulfil a particularly important role in these situations, as the alternative might be that people do not access health services at all.^13^ The ability to utilise self-care interventions that are available, accessible, acceptable, and of good quality is a core component of promoting and protecting the right to health. Furthermore, ensuring availability, accessibility, acceptability and good quality information, goods and services should form the foundation of relevant laws, policies and regulations. It is critical to balance the importance of quality and safety against the challenge of not restricting access. Attention is also required to promoting participation of users, non-discrimination, informed decision-making, privacy and confidentiality, and accountability. When human rights considerations are addressed, users’ exercise of their rights to health, to information, and to autonomous decision-making can significantly improve. However, for people who are unable to defend their right to health, improved health coverage and increased access to self-care interventions for SRH can only happen within the context of a safe and supportive enabling environment. For instance, self-care strategies such as HIV self-testing are highly acceptable to lesbian, gay, bisexual, and transgender (LGBT) persons who experience stigmatisation and corresponding SRHR access barriers.

The scoping process for WHO normative guidelines is the process of defining what the guideline will include and what it will not include.^14^ Qualitative reviews conducted to complement systematic reviews that inform guidelines can be further strengthened by the lived experiences of those whom the recommendations are intended to affect. Including this feedback within normative guidance development provides key insight into the understanding, opinions, challenges and opportunities regarding, in this instance, self-care interventions. The example of the global values and preferences survey conducted prior to updating WHO Guidelines on SRHR of women living with HIV is a valuable example of meaningful community engagement in the development of normative guidance and also revealed the extent of gender-based violence and mental health challenges experienced by women living with HIV, and the impact these have on their lives.^15,16^ Given that the implementation of self-care interventions for SRHR would affect components of UHC such as health service delivery systems, the health workforce, governance and legislation, and given also the myriad of such interventions available or in the development pipeline, there has been insufficient research exploring perceived benefits to and concerns of laypeople and healthcare providers. This cross-sectional web-based study aimed to elicit perspectives on self-care interventions for SRHR to inform WHO guidelines. This paper aims to share the open-ended responses regarding access, acceptability, and implementation considerations.

## Methods

### Participant recruitment and data collection

A series of WHO expert consultations to inform the development of the consolidated guideline on self-care interventions included the development of a survey on the perspectives of healthcare providers and laypersons with experience and expertise in SRHR on awareness of, access to, preferences and concerns on self-care interventions for SRHR. Data was collected via an online survey between July 2018 and November 2018. The survey was hosted on the website of the WHO Department of Reproductive Health and Research (now the Department of Sexual and Reproductive Health and Research) and shared through a range of relevant listservs (Table 1). Open text boxes were placed in four sections of the survey to elicit perspectives on self-care SRHR interventions regarding: potential benefits, current concerns, preferred conditions for implementation, and future issues for consideration. All participants provided written online informed consent before being permitted to access survey questions. We obtained Research Ethics Board approval from the University of Toronto, Toronto, Canada (Protocol 36022).

**Table 1.** List of organizations and listservs to whom GVPS was disseminated

	Name of the organization/listserv	Date sent
**1**	WHO self-care SRHR ethical and social concerns Brocher meeting participants	2018-07-13
**2**	HRP/ RHR	2018-07-13
**3**	IBP initiative	2018-07-13
**4**	PMNCH Adolescent and Youth Constituency	2018-07-13
**5**	WHO self-care SRHR intervention scoping review meeting participants	2018-07-13
**6**	WHO Young health professionals meeting participants	2018-07-13
**7**	UNAIDS Youth Constituency	2018-07-13
**8**	Restless Development	2018-07-13
**9**	IFMSA – International Federation of Medical Students’ Associations	2018-07-13
**10**	FP2020	2018-07-13
**11**	Youth Coalition on Sexual and Reproductive Rights	2018-07-13
**12**	IPSF – International Pharmacy Students Federation	2018-07-13
**13**	SVRI	
**14**	AWID Newsletter	
**15**	Reproductive HIV listserv	
**16**	IFFS – International Federation of Fertility Societies	2018-07-13
**17**	WRI women’s research initiative	2018-08-29
**18**	WHO website	2018-08-25
**19**	Sex workers of Winnipeg Action Coalition	2018-10-12
**20**	Sex workers Education and Advocacy Taskforce	2018-10-12
**21**	Sex Workers Outreach Project Los Angeles	2018-10-12
**24**	African Sex Workers Alliance	2018-10-12
**25**	The Sex Workers Project	2018-10-12
**26**	Global Network of Sex work Projects	2018-10-12
**27**	Sex Worker Advocacy and resistance movement	2018-10-12
**28**	Caribbean LGBT Community (Facebook group)	2018-10-18
**29**	LGBT Argentina (Facebook group)	2018-10-20
**30**	Orgullo LGBT Argentina (Facebook group)	2018-10-24
**31**	Be Positive (Facebook page)	2018-10-24
**32**	OUT LGBT Well-Being (Facebook page)	2018-10-24
**33**	Marsa Sexual Health Center (Facebook page)	2018-10-24
**34**	Women’s Global Network for Reproductive Rights (WGNRR) (Facebook page)	2018-10-24
**35**	PinkDot SG (Facebook page)	2018-10-25
**36**	The Asian-Pacific Resource & Research Centre for Women (ARROW) (Facebook page)	2018-11-01
**37**	Women for a Change Cameroon	2018-11-09
**38**	AfA Singapore – Action for AIDS	2018-11-14

### Data analysis

Survey respondents were asked to share their thoughts on self-care SRHR interventions through open text boxes where they could provide qualitative written responses. Open-ended survey comments were extracted from Qualtrics directly into Microsoft Excel, where we analysed the data using NVivo 10 data analysis software. We applied a thematic approach to data analysis where three persons coded the open-ended responses. As a theoretically flexible approach, thematic analysis involves having multiple readers examine the data several times to identify both inductive (e.g. social and psychological support recommendations) and deductive (e.g. stigma as a barrier to SRHR care) themes. While reviewing the data, we took notes and developed preliminary codes, that we further refined into themes. These themes were subsequently organised into a thematic map separately for lay respondents and healthcare providers. Further information on the methodology and data analysis is available in the WHO global values and preferences report.^17^

## Results

### Demographics

**Figure 1.**
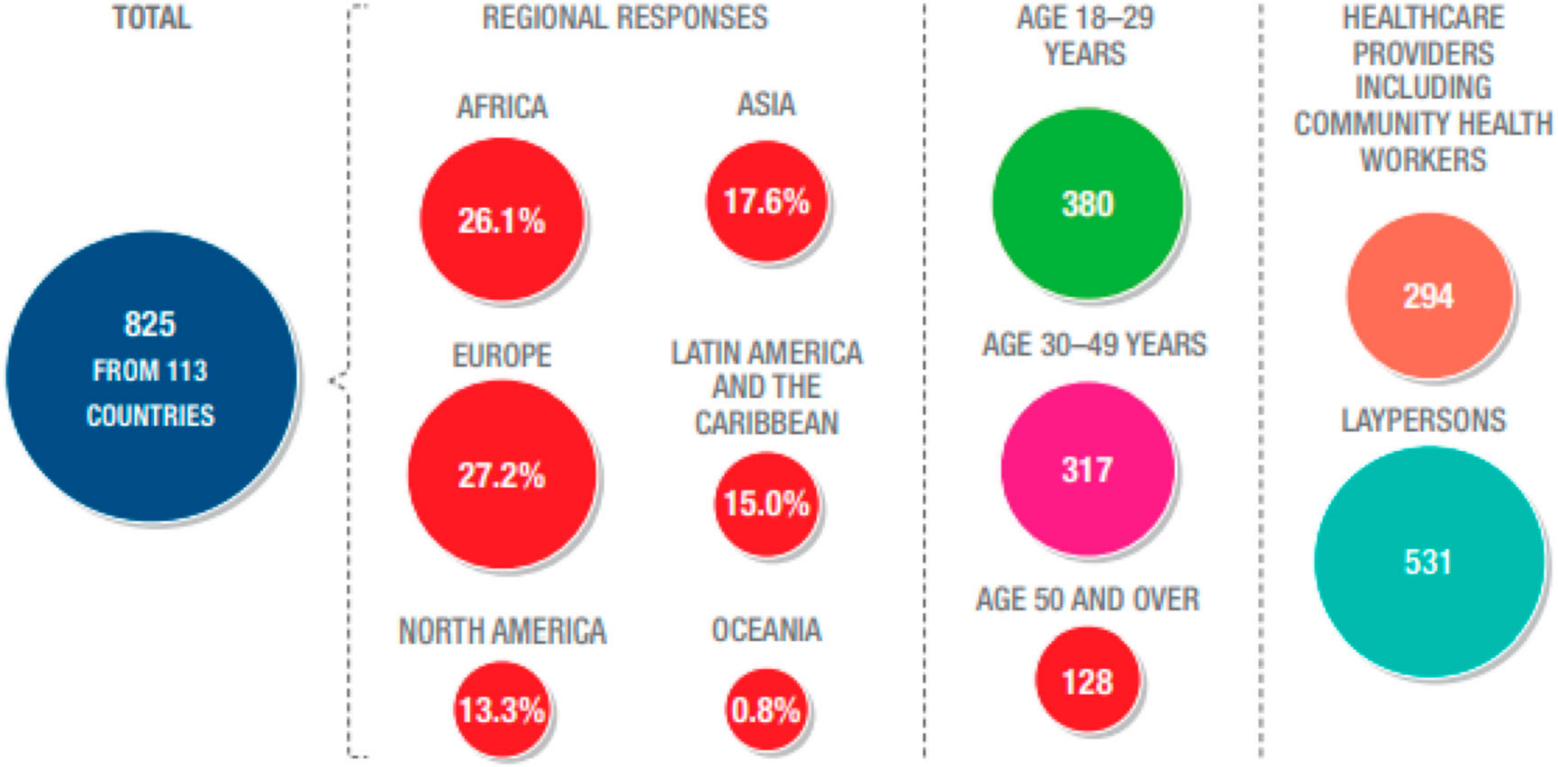
Number and selected characteristics of respondents to the global values and preferences survey on self-care interventions for SRHR

**Figure 2.**
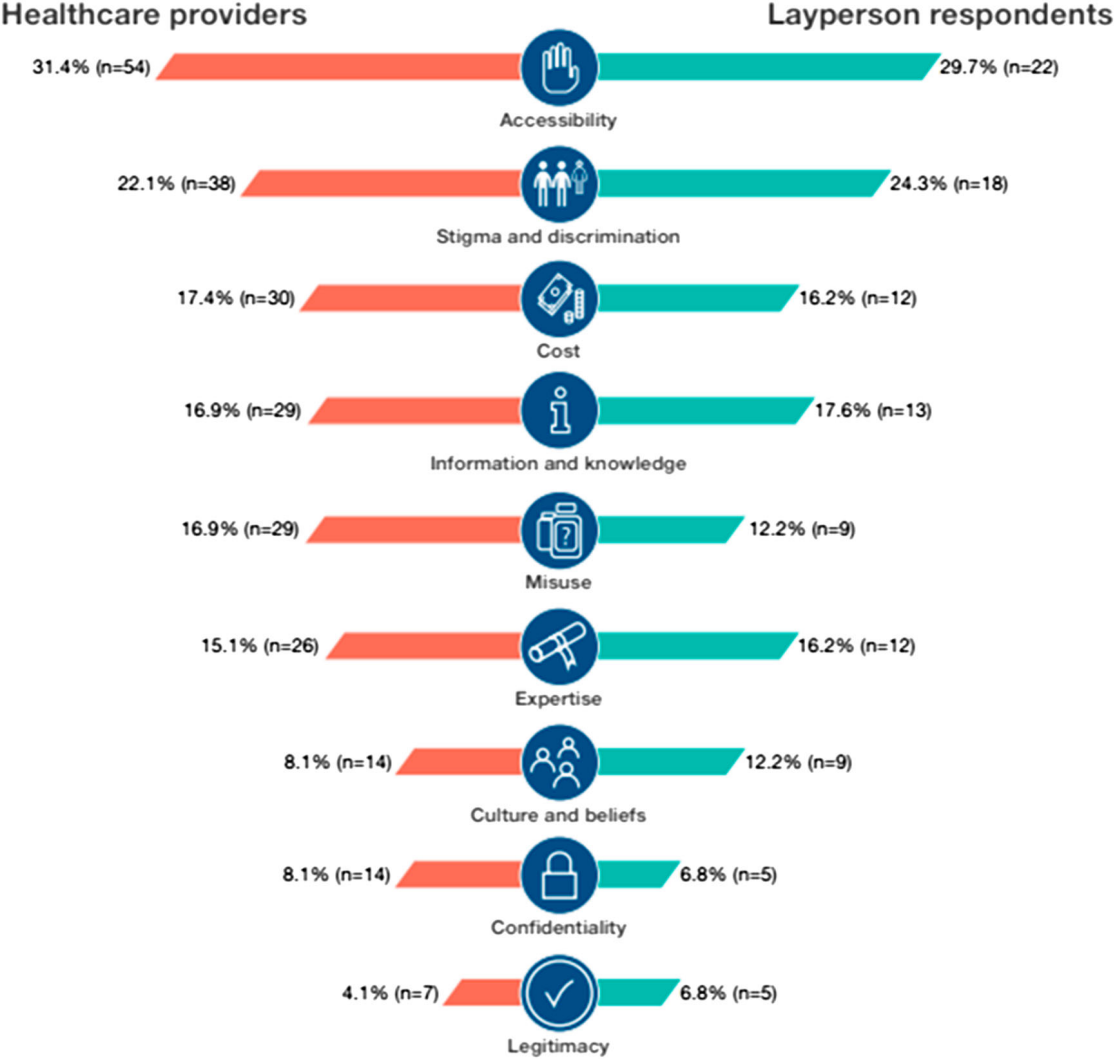
Healthcare providers and layperson responses per key outcome of the GVPS

This survey received 837 responses, from both healthcare providers (HCP; 43%, n = 360), and lay persons (57%, n = 477).^17^ Figure 1 shows the number of respondents per region and Figure 2 illustrates the main self-care interventions topics covered in this survey.

This manuscript focuses on the 326 participants who provided any qualitative responses to the open-ended questions, and the characteristics of the participants included in this qualitative analysis are presented in Table 2. Among these qualitative respondents, 78.3% (n = 252) were healthcare providers and 21.7% (n = 70) were not healthcare providers, and we identify them as “lay persons” (four persons did not specify employment status). Participants, mostly women (66.9%), were from 77 countries across the WHO regions, including the Africa Region (34.5%), Region of the Americas (32.5%), South-East Asia Region (5.6%), European Region (19.8%), Eastern Mediterranean Region (4.8%), and the Western Pacific Region (2.8%).^1^

**Table 2.** Characteristics of lay persons and healthcare provider participants who provided qualitative feedback in a global survey regarding self-care interventions for sexual and reproductive health and rights (n = 326)

Socio-demographics for qualitative respondents	Healthcare providers (n = 252)	Lay Respondents (n = 70)	All Respondents (n = 326*)
**Gender**			
Woman (self-defined; transgender and cisgender inclusive)	67.1% (169)	66.7% (46)	66.9% (216)
Man (transgender and cisgender inclusive)	32.5% (82)	30.4% (21)	32.2% (104)
Transgender	0.4% (1)	2.9% (2)	0.9% (3)
**Age**			
18–29	28.7% (72)	50.7% (35)	33.0% (107)
30–39	27.5% (69)	20.3% (14)	26.2% (85)
40–49	21.5% (54)	15.9% (11)	20.4% (66)
50–59	13.9% (35)	10.1% (7)	13.3% (43)
60–69	5.6% (14)	2.9% (2)	4.9% (16)
70+	2.8% (7)	0% (0)	2.2% (7)
**World Health Organization Region**			
African Region	34.5% (87)	23.2% (16)	32.6% (106)
Region of the Americas	32.5% (82)	27.5% (19)	31.4% (102)
South-East Asia Region	5.6% (14)	4.3% (3)	5.2% (17)
European Region	19.8% (50)	23.2% (16)	20.3% (66)
Eastern Mediterranean Region	4.8% (12)	5.8% (4)	4.9% (16)
Western Pacific Region	2.8% (7)	15.9% (11)	5.5% (18)
**Sexual Orientation**			
Heterosexual/Straight	85.3% (214)	73.9% (51)	83.0% (268)
Sexually Diverse (LGBQ+)	12.4% (31)	26.1% (18)	15.2% (49)
Prefer not to say	2.4% (6)	0% (0)	1.9% (6)
**Size of City/Town**			
A big city (above 1 million inhabitants)	48.0% (121)	55.7% (39)	49.7% (160)
A large city (300,000 – 1 million inhabitants)	18.7% (47)	18.6% (13)	18.6% (60)
A city (100,000 – 300,000 inhabitants)	10.3% (26)	4.3% (3)	9.0% (29)
Large town (20,000 – 100,000 inhabitants)	11.9% (30)	8.6% (6)	11.2% (36)
Town (1000 – 20,000 inhabitants)	7.5% (19)	7.1% (5)	7.5% (24)
Small town or hamlet (less than 1000 inhabitants)	3.6% (9)	5.7% (4)	4.0% (13)
**Highest Level of Education**			
Completed high school	6.0% (15)	21.7% (15)	9.3% (30)
A university bachelor’s degree	28.6% (72)	44.9% (31)	32.0% (103)
A graduate degree	64.7% (163)	31.9% (22)	57.8% (186)
Other	0.8% (2)	1.4% (1)	0.9% (3)
**Employment Status (all that apply)**			
Full time paid employment	64.1% (161)	54.3% (38)	62.0% (199)
Part time paid employment	13.5% (34)	5.7% (4)	11.8% (38)
Self-employed	10.0% (25)	4.3% (3)	8.7% (28)
Unemployed	2.8% (7)	2.9% (2)	2.8% (9)
Currently a student	13.9% (35)	32.9% (23)	18.1% (58)
Casual labour	1.2% (3)	2.9% (2)	1.6% (5)

*Four respondents did not report if they were healthcare providers or lay people.

### Perceived benefits of self-care interventions for SRHR

Among lay persons participating, self-care SRHR interventions were described as beneficial for reducing stigma and discrimination, increasing confidentiality and decreasing barriers to access. Among healthcare provider participants, perceived benefits included empowerment, increased uptake of SRHR services, and privacy, reducing exposure to stigma. Table 3 details the themes by respondent (lay person, healthcare provider) with illustrative quotations.

**Table 3.** Overview of themes regarding self-care interventions for SRHR from lay persons and healthcare provider participants of a global survey (n = 326)

Theme regarding self-care interventions	User perspective	Healthcare provider perspective
Benefits	Less stigma: “I think an option for self-initiated interventions is good to reduce stigma and discrimination.” (41-year-old woman, Uganda) Reduced barriers to access: “Going to a health care provider can be a major barrier for women to access reproductive health services. Putting care directly into the hands of women to manage is an important way to overcome this barrier” (29-year-old woman, United States)	Empowerment: “This can lead to improvement of knowledge and confidence.” (65-year-old man, Kenya) Increased uptake of SRHR services: “Greater uptake and adherence of interventions if self-initiated” (59-year-old woman, United States) Privacy and reduced stigma: “I think that having these interventions easily accessible and without stigmatization or shame would make these interventions easier, more pleasant and safer” (21-year-old woman, Poland)
Concerns	Stigma: “Young people are exposed to ill treatment by the doctor” (22-year-old man, Turkey) Accessibility concerns: “Confidentiality is a problem when you go to access services” (56-year-old woman, Liberia) Trustworthiness of intervention: “I would want to make sure they are trusted, not counterfeit, if I buy them online.” (35-year-old woman, United States) Potential for misuse: “an intervention led by a doctor or health care provider is safer and maintains safer reproductive health” (63-year-old woman, Pakistan)	Insufficient user knowledge: persons would have “*incomplete information to make a really informed choice”* (43-year-old woman, Spain) Stigma: there is the need for “*accessibility to self-interventions without prejudice”* (32-year-old man, Nigeria) Accessibility concerns: people experience “*lack of access to health services when needed even if they exist due to many barriers”* (56-year-old woman, Italy) Side effects/ complications: concerns regarding “*handling of complications”* (54-year-old man, Kenya)
Implementation considerations	Knowledge and information: “Self-initiated interventions requires awareness, good educational background and community participation” (70-year-old man, India) Accessible interventions and linkages to healthcare: “Some interventions need counselling before they are used, how will that happen in self-initiated interventions?” (44-year-old woman, Uganda)	Healthcare linkages: “this should be an integral part of all interventions with clear guidance provided on all platforms of how to access services if needed” (56-year-old woman, Italy) Increased community engagement: “spreading information about that in the community” (21-year-old woman, Poland)

#### Lay perspectives of perceived benefits of self-care interventions for SRHR

Respondents identified self-care SRHR interventions as:

- potential solutions to barriers they face in realising optimal SRHR:
  “I think that having these interventions easily accessible and without stigmatization or shame would make these interventions easier, more pleasant and safer.” *(21-year-old, Poland)*
  “I believe self-care interventions can play a huge role in reducing barriers to accessing services due to stigma associated around it, especially in Sub Saharan Africa.” *(26-year-old, Uganda)*
- solutions to potential access barriers because of their convenience and low cost:
  “I think it’s really important to have self-care interventions available over the counter at pharmacies at a low cost so that people can access them without having to visit a healthcare provider, which adds an additional cost in both monetary value and lost time. Oftentimes going to a healthcare provider to access some of these self-care options makes women feel shameful and some healthcare providers don’t do a great job of making it a safe/empowering space for women to choose the method that is right for them. It would be amazing to have more options available/more easily accessible to women in the United States.” *(29-year-old, USA)*
  “Self-initiated interventions are important because it reduces stigma both in the community and family, it is less cost because there is no service fee for the health provider and accessibility is good because it can be kept at home and you can use any time.” *(38-year-old, Malawi)*
- empowering to users, including marginalised populations, by providing opportunities for users to make their own SRHR choices:
  “Self-care interventions increase access and empowerment, reduce cost and stigma and put decision making in women’s/men’s hands.” *(42-year-old, United Kingdom)*
  “Access would be the most important. While I don’t have concerns visiting a doctor or health centre as I am out and comfortable with my sexuality, those who are not out would be unlikely to access a health service and disclose their sexuality to a doctor.” *(38-year-old, Thailand)*

#### Healthcare provider perceptions of benefits of self-care interventions for SRHR

Healthcare providers noted that self-care interventions for SRHR promote empowerment and self-confidence, leading to informed decision-making, including increased power to people (54-year-old, Switzerland); and improving people’s autonomy (33-year-old, Ecuador), including the importance to empower the community with information about their SRHR (58-year-old, Kenya). Some described the personal benefits of self-care interventions to marginalised populations involving a mix of increased convenience and access as it enables them to make informed decision without being pressured by anyone (25-year-old, Kenya) and [these interventions] remove gatekeepers (56-year-old, New Zealand).

Self-care SRHR interventions were also described as having the potential to increase the population’s knowledge and information on SRHR, which benefits their health and well-being in multiple ways:

“Self-testing could revolutionise disease detection within the public health sphere. With the right amount of support channels this could empower people to take ownership of their health. There has been a lot of negative flak around self-testing, but I feel that to empower people is the rationale of thought leadership practices, which could be successful for public health initiatives.” *(28-year-old, South Africa)*

Another commonly reported benefit was increased uptake, with respondents noting how self-care interventions were especially beneficial for populations who may not engage in SRHR services as often as needed. This includes adolescents and young people: “*Many [interventions] are excellent for increasing access by youth to SRH services as they are often deprived of knowledge and resources*” (56-year-old, Italy). Participants also discussed how self-care interventions result in time savings that could also increase uptake as well as faster treatment if patient don’t have time to wait in health clinics and hospitals. (31-year-old, Serbia) Self-care strategies were identified as having the potential to both improve health and ease burdens on the healthcare system: “*will assist in easing the public healthcare system load and encourage health seeking behavior of the public”* (30-year-old, Philippines).

Healthcare provider respondents also noted that patients are better able to maintain their privacy and confidentiality with self-care SRHR interventions. This was described as especially beneficial to vulnerable populations such as women in abusive relationships: “*Privacy [is a benefit], especially for women in abusive situations where husbands may control medical care”* (40-year-old, Canada). Another healthcare provider described the ways that stigma and discrimination would shape her own personal decision in using self-care interventions: “*If the intervention is normal and accepted by the society then it’s fine, however, if it would lead to people making judgements then I would prefer to do it myself”* (23-year-old, Qatar).

### Concerns regarding self-care interventions for SRHR

Some concerns were shared across participants, whereby lay respondents and healthcare providers were both concerned about barriers such as stigma and accessibility issues. Lay respondents also brought up concerns regarding the trustworthiness of the intervention and potential for misuse, while providers discussed insufficient user knowledge and issues regarding side effects or complications.

#### Lay concerns regarding self-care interventions for SRHR

Barriers to self-care SRHR interventions identified included stigma and confidentiality. For instance, a participant described “*Non-judgmental and quality care and service [as] the paramount consideration when one decides feeling self-initiated or health service provider assisted SRH service”* (30-year-old, Philippines). Another participant narrative reflected this: “*Stigma in Nigeria is still a huge issue. I'd like for more discreet means of accessing self-initiated interventions in such countries”* (25-year-old, Nigeria).

Similarly, the overreliance on healthcare professionals for self-care interventions for SRHR was identified as a barrier:

“The field is too medicalized. Clients have been taught to go to the doctor for everything. It will take time and effort for people to fight for and access some of these interventions without the use of a healthcare provider.” *(31-year-old, USA)*

Another concern related to self-care interventions was the question of trustworthiness and quality of products. Constrained access to quality, safe, and effective medical products creates a vacuum that is too often filled by substandard and falsified products – which are authorised medical products that fail to meet either their quality standards or their specifications, or both.^18^ This issue was identified as a potential concern for products accessed online or through pharmacies: “*Private drug shops, clinics need to be well regulated and could be sources of more burden and challenges”* (46-year-old, Uganda), and “*Some girls may need emergency contraception after unprotected sex, but they may buy it online due to other's attitudes and the drugs online are not really safe”* (19 year-old, China).

A final concern was the potential for misuse. Some respondents described a concern over complications and safety-related consequences that may arise from misuse of a self-care product and/or due to poor health literacy: “*[T]here is a risk for the general public of abuse of misuse due to lack of knowledge. There is a lot of misinformation out there so there must be safeguards and some control”* (56-year-old, Italy). Others explained that, in addition to poor knowledge levels, a lack of current regulations would pose problems: “*Feeling self-initiated interventions are bound to be abused, poorly regulated and policies are not very strong to support these interventions”* (46-year-old, Uganda).

#### Healthcare provider concerns regarding self-care interventions for SRHR

Healthcare provider concerns fell into four themes: insufficient user knowledge, stigma, accessibility issues, and side-effects and complications. Concerns regarding knowledge and information centred on the need to ensure persons had accurate information about the self-care interventions they were using or planning to use: “*I think there is a lot of false information out there, and it can be hard to distinguish the credible sources from the ones that aren't”* (39-year-old, USA). The lack of appropriate information was perceived to have potential harmful consequences on healthcare decisions.

Stigma, including shame, community norms, and at the state level a lack of supportive policies, affected the experience of healthcare: “*shame accompanying using them, e.g. because of social stereotypes”* (21-year-old, Poland); as well as “*how sociocultural issues may exist on the macro level, through traditional and discriminatory state policy”* (33 year-old, Cameroon).

“*Accessibility, including high costs and difficulty of access are important issues for some methods”* (60-year-old, Brazil) was mentioned as particularly concerning for vulnerable populations, and ways to minimise this included making available “*non-prescription, over the counter, with an ability to seek additional guidance”* (37-year-old, USA).

Issues regarding side-effects and potential complications were also noted by healthcare provider respondents. Respondents expressed concern regarding how patients would respond to interventions that cause side-effects or potential harmful consequences: “*side effects [are a concern] and as result decision to quit and refuse the self-initiated interventions in the potential future”* (27-year-old, Moldova). Another respondent discussed the need to link persons to healthcare as needed following uptake of a self-care interventions: “*Access to and support provided by health services if the patient has used something incorrectly and needs treatment/support to correct that”* (54-year-old, Switzerland).

### Implementation considerations regarding SRHR self-care interventions

When asked about recommendations for implementing self-care interventions, lay provider responses encompassed concepts of (a) knowledge and information and (b) accessibility of interventions, as well as linkages to healthcare. Healthcare provider responses also discussed strategies for creating linkages to care, as well as the need for community engagement.

#### Lay recommendations for SRHR self-care interventions

Many respondents explained that being well-informed, based on information from trustworthy sources, was important to inform their choice: “*I just want to be well informed of the interventions and choices I have, and then I want to choose by myself without needing a doctor”* (23-year-old, Portugal). Respondents also explained how this need for information, in addition to other ideal qualities, extends to the usage of self-care interventions. For instance, “*Convenience, minimal discomfort (pain), cost and user-friendliness are top-most considerations for me when choosing such products or accessing such services”* (42-year-old, Nigeria) explained important criteria for these interventions.

Accessibility included affordability of self-care interventions, as well as access to healthcare provision as needed: “*The interventions should be readily available and at minimal cost”* (30-year-old, Kenya). Respondents specified that these interventions should either be offered for free, at low cost for consumers (e.g. covered by insurance or universal healthcare), or on a sliding scale. When needed, respondents described how health service linkage should ensure access to equitable, informed services. For instance, a respondent noted that individuals who are sexually diverse have additional considerations when seeking out SRHR resources, noting that they would access services “*As long as wherever I am getting information is safe, ethical, accessible and queer friendly”* (25-year-old, Australia). One respondent described that healthcare provider expertise is not always guaranteed: “*It depends if I feel it’s something I can get sufficient information on without seeing a doc. But so many are poorly informed on sexual health and, generally, access to the intervention”* (34-year-old, Mexico). Others described possible complications regarding poor accessibility: “*I just hope they don’t eliminate the need of a healthcare provider because really, anything could go wrong really”* (24-year-old, Kenya). The importance of healthcare provider engagement shifted depending on the intervention. Some described why particular interventions may need more healthcare provider involvement:

“Simple interventions can be feeling self-initiated like birth control or morning after pills or self-testing. But abortion pills, HIV medication pre and post exposure, STI medication should be through a health provider because of complications arising from the condition or from the medication taken.” *(48-year-old woman, Kenya)*

Access to counselling along with self-care interventions emerged as important: “*there should be very strong counselling communication on the need to consult a doctor or other healthcare worker should the client both be happy with results or if they should develop complications”* (58-year-old, Democratic Republic of Congo).

#### Healthcare provider recommendations for SRHR self-care intervention implementation

Two overarching themes from healthcare provider recommendations for SRHR self-care strategies included healthcare linkages and community engagement. First, healthcare providers suggested strategies for linking self-care interventions for SRHR to the healthcare system as needed. Common suggestions included providing healthcare provider contact information, a referral directory system, increased education, mobile apps, use of support, and instructions on next steps. Respondents explained different strategies for these linkages, such as “*involving health workers before commencement to ensure linkages”* (49-year-old, Nigeria). Participants suggested providing clear instructions to outline how to proceed following use of interventions to link with healthcare if needed “*by outlining steps to follow after each test results either negative or positive”* (32-year-old, Nigeria). Many highlighted the benefits this might have for vulnerable populations: “*There needs to be a directory of clinics/doctors/healthcare providers that are sensitive and not prejudiced against people who need healthcare services for SRH problems. Patients/clients could be matched to the nearest doctor/clinic through a web application”* (24-year-old, Turkey).

The heterogeneity within self-care interventions also meant that some strategies had very low risk, reducing the likelihood of needing to interface with the health system: “*In case of any complications or adverse reactions, unproper use of the intervention, if the risk is minimal and the supervision of the health provider is not required, then let it be”* (27 year-old, Moldova).

Healthcare provider respondents also discussed the need to increase education to ensure linkages to care. This was explained as being done through raising awareness, knowledge levels, and health literacy among the population. Some suggested the use of the media and prominent public figures, while others thought increasing education on the community level would be most effective: “*More awareness and more media coverage could help”* (26 year-old, Nepal). This was identified as helpful for reducing the impact of negative community beliefs on self-care SRHR interventions; as well as the need for “*education for community leaders to mitigate sociological, cultural, religious factors”* (71 year-old, USA).

The use of social and psychological support was described as another option to ensure linkages to care, including counselling, psychological care, and guidance: “*A toll-free number that provides quality counselling services should be put on the products used in the interventions so that people can easily talk to someone who will guide them on what to do”* (24-year-old, Uganda).

Support was also discussed as potentially building on community resources. For instance, some respondents mentioned that community health workers may be well positioned to provide support: “*Clients need assurance of confidentiality and trust in the personnel. Thus, through the use of professional and empathetic community health workers”* (32-year-old, Cameroon). Community-based strategies were also suggested as important for sharing information and knowledge regarding self care and increase uptake: “*Community based patient education rather than health facility-based heath talks will drive uptake of the self-initiated care better because oftentimes, non-utilisation is often due to lack of or inadequate awareness or knowledge about them”* (42-year-old, Nigeria).

## Discussion

WHO recognises the value and potential contribution of self-care interventions within health systems, and the rapid advances being made in quality, evidence-based health care, health-seeking practices, and health information that can be initiated by individuals. In line with the WHO conceptual framework for self-care interventions, there are two, complementary pathways of change to improve health and well-being. This includes: (1) increasing autonomy and agency through empowering individuals, particularly vulnerable populations, to advance their SRHR; and (2) transforming a health systems approach to create safe and supportive enabling environments that are open to and support and serve vulnerable populations. In line with the process for the development of the WHO global normative guidance on self-care interventions, continued engagement of healthcare providers as well as the self-carers and care-givers has the potential to transform *ad hoc* activities into coherent policies and programmes for implementation that improve SRH, human rights and UHC.

Lay persons and healthcare providers from 77 countries shared perspectives on benefits and concerns regarding use and uptake of self-care interventions for SRHR, as well as implementation considerations. Overall, perspectives were remarkably similar between lay persons and healthcare providers. Both groups perceived the potential of these strategies to reduce stigma, discrimination and other access barriers, in turn increasing coverage. Lay persons shared concerns regarding intervention trustworthiness and the potential for misuse, while healthcare providers shared worries of insufficient user knowledge, health literacy and challenges managing potential side effects and/or complications. Participants detailed considerations for improving knowledge, access, healthcare linkages and community engagement.

Similar to prior research,^19–21^ participants discussed self-care SRHR strategies as important for enhancing access for marginalised communities. Meaningful engagement of lesbian, gay, bisexual, and transgender persons, adolescents, women, and persons with disabilities were highlighted as important to include in implementation to improve health coverage. Stigma and discrimination emerged as important themes across elements of this study. While participant narratives corroborated research positing that self-care strategies can help persons to mitigate stigma and discrimination by improving privacy,^6,7^ both lay persons and healthcare providers noted that stigma and discrimination toward communities and toward SRH could be a persistent barrier to access. These findings suggest the need for integrated stigma and discrimination mitigation strategies alongside self-care SRHR implementation. Also aligned with prior research, participants noted that these strategies could foster agency and empowerment.^22,23^

**Figure 3.**
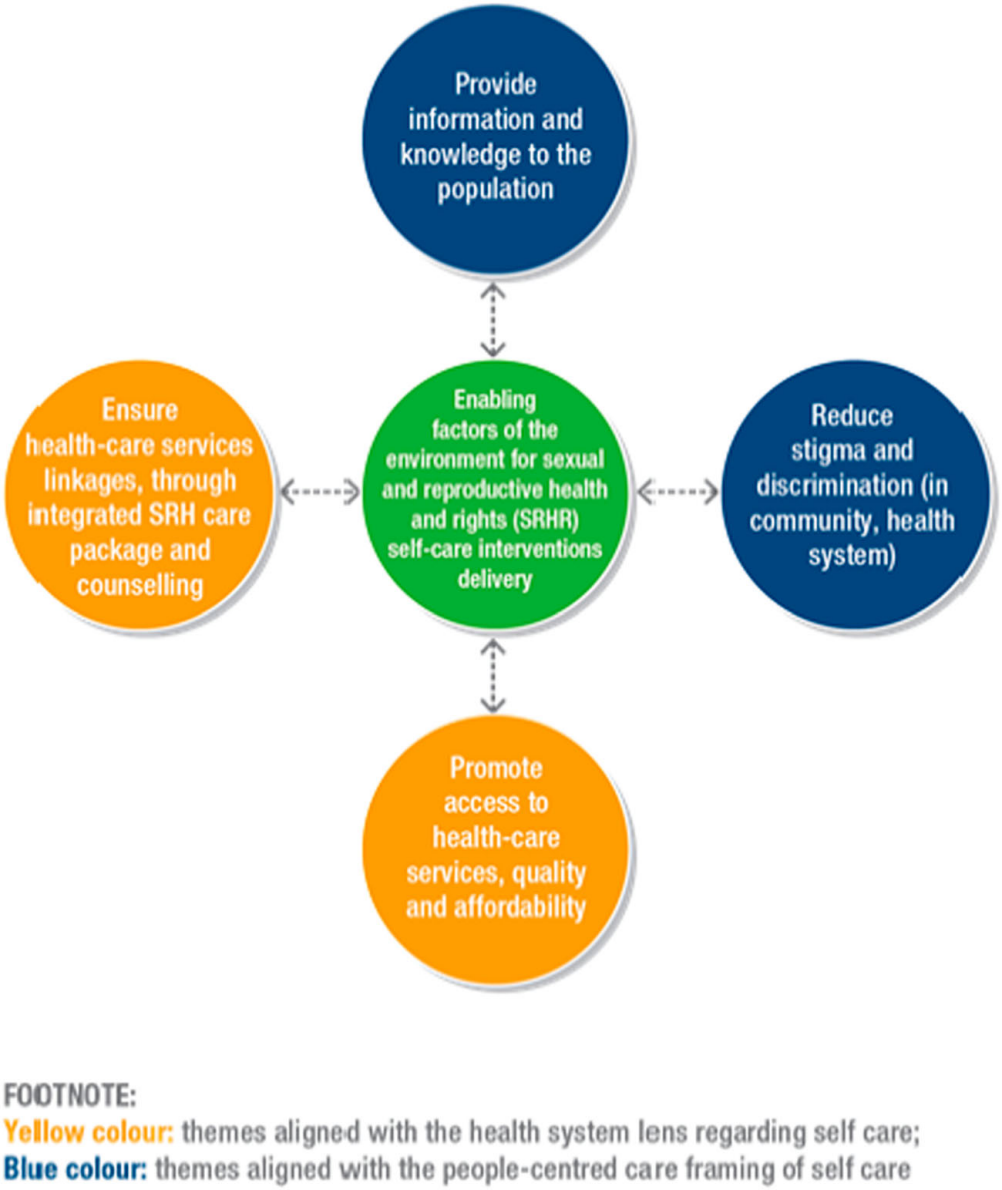
Health systems and people-centred strategies to support self-care interventions for SRHR

Based on the outcomes of the survey, we developed the conceptual framework in Figure 3 to depict the ways in which findings emerged at both the people-centred care^9^ and systems levels.^4^ For instance, the focus of the *people-centred care*^9^ approach on agency and capacity building is evidenced in discussions regarding the need to provide information on self-care interventions, and the potential of this knowledge to be empowering. Empowerment is also connected with reducing stigma and discrimination in community and health systems in order to increase access, respect and protection of human rights. The focus of the *systems level*^4^ on optimising health and linkage to care^11,12^ emerged in themes regarding the strategies for linkage as well as the need for accessible, affordable interventions and healthcare services, including psychosocial support. These findings align with the conceptual framework^4^ for self-care interventions that includes the need for trained healthcare providers, psychosocial support, information, and freedom from stigma and discrimination.^4^ In Figure 3, we highlight strategies for the health systems themes in yellow, the people-centred themes in blue, and the centre – the enabling environment for self-care interventions for SRHR – as green to show their intersection.

There are study limitations. This was a non-random convenience sample accessed via WHO website and global SRH and HIV listservs, thus the sample may over-represent persons interested in, and knowledgeable about, SRHR. The findings may therefore not be globally generalisable. Second, while there is a large geographic spread of participants, there are insufficient responses per country or region to conduct a more focused analysis of regional similarities and/or differences. Further research is needed to elicit context-specific priorities. Third, the open-ended responses were about self-care interventions generally, rather than about a specific intervention (e.g. HIV self-testing), hence the findings can be considered as a snapshot of considerations to inform future research and implementation that will need to delve into the heterogeneity of interventions. Fourth, the survey was conducted in English, French and Spanish due to budgetary and time restrictions; future research is needed to include a range of languages to increase access. Finally, the online nature of the survey limited access to participation for persons without regular access to a phone/computer and/or internet. These limitations point to the need for research to elicit a wider range of perspectives on self-care SRHR interventions. Although the data is not globally generalisable, the survey aimed to elicit perspectives to supplement systematic reviews, that included qualitative reviews, for the development of new WHO recommendations and good practice statements on self-care interventions for SRHR. Despite these limitations, to our knowledge this is the largest survey to date exploring global perspectives of self-care interventions for SRHR that included both lay person and healthcare provider perspectives from a range of geographical contexts creating a shared knowledge with a very interesting common ground around the globe.

To better understand the effects of self-care interventions in people’s lives, implementation strategies need to be linked to clear outcomes. Successful mainstreaming of self-care interventions will therefore require monitoring and evaluation early on. While monitoring and evaluation is common practice for programme implementation of focussed health topics or interventions, such as the number of antenatal care visits for maternal health or use of antiretrovirals for HIV treatment, it is far less common in domains where policies and programmes are aimed at an organisational change. Introduction of quality self-care interventions is a true paradigm shift in the way health care is delivered. Its potential to bridge people and communities through primary health care to reach UHC is underexplored. Moving forward, researchers, policy makers, and practitioners can consider the participant narratives regarding the need to consider both the heterogeneity of self-care interventions for SRHR as well as the needs and lived experiences of diverse populations. These lay persons’ and healthcare providers’ perspectives underscore the urgent need to increase access, reduce stigma and discrimination, and improve knowledge of self-care SRHR interventions to increase UHC in order for individuals and communities to realise their SRHR and for countries to achieve the Global Development Goals.

## Footnotes

1 The proportions refer to the subset of the sample who provided any qualitative response, so these numbers are different from those for the survey as a whole.
